# Research approvals iceberg: how a ‘low-key’ study in England needed 89 professionals to approve it and how we can do better

**DOI:** 10.1186/s12910-018-0339-5

**Published:** 2019-01-25

**Authors:** Mila Petrova, Stephen Barclay

**Affiliations:** Primary Care Unit, Department of Public Health and Primary Care, University of Cambridge, Institute of Public Health, Forvie Site, Robinson Way, Cambridge, CB2 0SR UK

**Keywords:** Ethics committees, Research [MeSH], Ethical review [MeSH], Ethics, Research [MeSH], Bioethics [MeSH], Institutional review boards, IRB, Biomedical ethics, Evaluation studies [MeSH]

## Abstract

**Background:**

The red tape and delays around research ethics and governance approvals frequently frustrate researchers yet, as the lesser of two evils, are largely accepted as unavoidable. Here we quantify aspects of the research ethics and governance approvals for one interview- and questionnaire-based study conducted in England which used the National Health Service (NHS) procedures and the electronic Integrated Research Application System (IRAS). We demonstrate the enormous impact of existing approvals processes on costs of studies, including opportunity costs to focus on the substantive research, and suggest directions for radical system change.

**Main text:**

We have recorded 491 exchanges with 89 individuals involved in research ethics and governance approvals, generating 193 pages of email text excluding attachments. These are conservative estimates (e.g. only records of the research associate were used). The exchanges were conducted outside IRAS, expected to be the platform where all necessary documents are provided and questions addressed. Importantly, the figures exclude the actual work of preparing the ethics documentation (such as the ethics application, information sheets and consent forms). We propose six areas of work to enable system change: 1. Support the development of a broad range of customised research ethics and governance templates to complement generic, typically clinical trials orientated, ones; 2. Develop more sophisticated and flexible frameworks for study classification; 3. Link with associated processes for assessment, feedback, monitoring and reporting, such as ones involving funders and patient and public involvement groups; 4. Invest in a new generation IT infrastructure; 5. Enhance system capacity through increasing online reviewer participation and training; and 6. Encourage researchers to quantify the approvals processes for their studies.

**Conclusion:**

Ethics and governance approvals are burdensome for historical reasons and not because of the nature of the task. There are many opportunities to improve their efficiency and analytic depth in an age of innovation, increased connectivity and distributed working. If we continue to work under current systems, we are perpetuating, paradoxically, an unethical system of research approvals by virtue of its wastefulness and impoverished ethical debate.

## Background

The gaze of scrutiny in the relationship between research studies and Research Ethics Committees (REC), Institutional Review Boards (IRB) and other bodies which grant permissions for (funded) research to proceed is typically unidirectional. Such organisations and their members review research studies for the ethical, legal and governance issues they raise. Occasionally, roles are reversed: research studies engage in probing and critical exploration of the work of those gatekeepers. This paper represents one such example. We build on existing literature and the striking findings of an audit-type study of the ethics and governance approvals of one of our recent projects (“*Prepared to Share?*: a study of patient data sharing in complex conditions and at the end of life” [1]) and offer proposals for change.

The paper is structured as follows:

An introduction sets the scene of researchers’ attitudes towards ethics and governance approvals processes.

We then present an extensive review of the empirical and critical literature on RECs, IRBs and ethics and governance approvals for health research (here understood to include biomedical research, health services research, and health-related research informed by the behavioural and social sciences and the humanities). We present a more detailed account of the background literature than typical of an empirical paper, as the literature is dispersed and little known. We include scholarly work which goes further than the mainstream storyline around the ethical regulation of research, namely how vital the work of ethics committees is to prevent a repetition of, for instance, the Nazi atrocities in the name of science, the Tuskegee syphilis experiment or experiments like those of Milgram and Zimbardo. The publications that transcend this narrative do not (obviously) question the need for ethical research, but probe into or challenge the extent to which the current systems are achieving that goal.

The respective scholarly field is far broader than appears at first sight, yet it lacks both ‘self-awareness’ as a distinctive area of study with core assumptions, questions, typical methods, exemplar studies, etc. and meaningful visibility to readers, at least outside the realm of bioethics. At the same time the work of RECs, IRBs and other permissions granting bodies affects all “human subjects research”. It is an oft-forgotten determinant of the contents and boundaries of our scientific knowledge.

Following the literature review, we present our empirical findings. These are from an audit-type study of the ethics and governance approvals of a project on patient data sharing at the end of life in a single locality in England. The trigger words of ‘data sharing’ and ‘end of life’ notwithstanding, this was, in the words of a research governance officer, a “low-key” (low-risk) study in terms of the ethical and governance issues it raised.

A further clarification of scope and terminology may be helpful. Most of the background literature addresses the ethics approvals of studies. However, study approvals are a broader enterprise, variably termed (at least in the UK) “research ethics and governance approvals”, “ethics and R&D (research and development) approvals”, “study assurances”, etc. Additional sign-offs are needed by R&D departments of participating organisations or university research offices (if the ethics approval has been granted by a committee unattached to a university) around compliance with regulations or the capacity of an organisation to host a project. Researchers involved in human subjects research often use “ethics approvals” as a synecdoche for this broader class of research ethics and governance approvals, not least because the latter are typically contingent on the former and because the bulk of the documentation is first prepared for the ethics review. Our paper concerns this broader class of approvals.

Finally, we outline six proposals for change in the systems of ethics and governance approvals, with potentially global application. Key features of these proposals are that they emphasise the enrichment of the ethical debate at least as much as the efficiency gains and that they rely strongly on opportunities provided by the growing sophistication and use of IT.

### The negative set point of researchers’ expectations

Most researchers conducting human subjects research soon internalise an emotional response to the REC/IRB process and the broader system of research approvals. While researchers variably like or dislike different phases of a study, we are still to meet one looking forward to ethics and governance approvals applications. More accurately, we are still to meet a researcher mentioning that they are about to embark on those processes without pulling a face or changing their tone of voice. They/we may latter comment that the process went smoothly, was not as bad as expected, and was even quite useful. In some cases, they/we may feel resentful about being ‘told’ what we can or cannot do when we think we ‘know better’. For fully valid or rather suspect reasons, the set point of researchers’ expectations of the ethics and governance approvals for research studies is often remarkably negative.

This emotional response may be more local, less generalisable than it appears to us, fostered by the systems and institutions we have been working in. We hope readers will be willing to join a debate, both on the scope of problems of research approvals and on new solutions to them from a variety of perspectives and experiences. It is, nonetheless, a sad state of affairs to be experienced even in pockets of the academic world. First, in research as in daily life, ethical dilemmas (typically the starting point of approvals after research has been funded) are amongst the most engaging and enlivening topics of conversation. Then, the people behind the bureaucracy are often thoughtful, well-intentioned individuals willing to make a difference to research and research subjects. REC and IRB board members are generally volunteers. Some of them are, or have been, researchers themselves. Many members of staff working on broader governance approvals, for instance in clinical research networks, individual healthcare organisations or universities, are consistently going the extra mile to support researchers in navigating complex systems. They can easily introduce inordinate delays or even bring the whole enterprise down if only ‘working to rule’ and within the explicit remit of their (paid) roles.

Yet for many researchers with no insider experience in such processes, ethics and governance approvals are hardly ever about ethical deliberation and motivating exchanges with people supporting you to conduct a better study. More often than not, researchers experience them as exercises of form filling; of engaging with a tiresome and wasteful bureaucracy; of learning the rules and language of a game and sticking with your prescribed roles in it; and, more cynically, of somebody out there caring about ethical conduct only insofar as litigation and reputational damage can be avoided. As a result, many of us working in areas or with methods that may be “exempt” from REC/IRB review would consider fitting a study into an exempt category and avoiding the process altogether, while upholding ethical research standards as an integral part of taking professional responsibility (more or less successfully).

Such impressionistic accounts of researchers’ discontent are borne out by formal studies. In a survey of social researchers working on health and applying for NHS ethics approval (UK system under the National Health Service), Richardson and McMullan [2] found that 78% of their respondents (101) agreed or strongly agreed that the time for preparing an application inhibited social research in health and 75% (98) either disagreed or strongly disagreed that the amount of paperwork required was reasonable. Over half (51%, 64 individuals) reported they had modified their research design for the worse in order to secure ethical approval; 45% (58) had modified their research design to avoid the process; 30% (40) had abandoned their research before applying and 12% (16) during the application process (though we cannot assume that the ethics approval process was the sole reason). Twenty-one percent (26) revised their research design after obtaining approval and did not seek further permissions. Fifteen percent (19) had started research needing NHS research ethics approval without applying and 12% (14) had commenced their research before the formal approval was given. More positively, 32% (41) of respondents had made changes for the better as a result of the ethics approval process [2].

Interestingly, for those of us who internalise such negativism, this happens almost entirely through socialisation in our research community and overlaying our personal experiences on the socialisation process. We do not learn to be critical of the ethics and governance approvals of human subjects research by reading academic and professional literature presenting critical arguments or worrying empirical findings. Such literature is sparse, dispersed and hardly ever a syllabus item.

### The sparse and fragmented literature on ethics and governance approvals

To our knowledge, there are only two well-known book-length critical explorations of IRBs (with the popularity still confined to bioethics circles rather than those of medical and health researchers): Zachary Schrag’s *Ethical Imperialism*: *Institutional Review Boards and the Social Sciences, 1965–2009* (2010) [3] and Laura Stark’s *Behind Closed Doors: IRBs and the Making of Ethical Research* (2011) [4]. Schrag’s *Ethical Imperialism* explores the regulation of research in the social sciences and humanities by IRBs and how derivative this regulation is of the concerns, ethical codes, rules and blueprints of legislation and regulation pertaining to biomedical and, to some extent, psychological research. It illustrates how limited or unsuccessful the involvement of social scientists in the debates was (with them practically “left howling outside the door” and becoming “objects of policy rather than its authors”, *op.cit.*, p. 4, p. 8). As any good history book, it shows the contingency of much of what we now take for granted and ethical in human subjects research (e.g. the very concept of prior/prospective review; the risks-benefits framework; even the “do not harm” principle once applied outside of patient populations, such as in studies aiming to increase public accountability). Much of the regulation wielding “totemic influence over the practice of research ethics” (*op. cit.*, p. 78) has also been shaped by time and staff limitations; the moment-to-moment availability or lack of particular types of expertise; by public and political pressures; by tactics and personalities; by the supposedly neutral work of redrafting and editing; by the gradualism of introducing changes, which keeps them off the radar of those most affected by them. Schrag’s book is an unlikely page-turner for work so meticulous in tracing and representing historical detail, so careful in crafting precise and information-dense sentences, and so concerned with getting the history right as opposed to advancing a righteous argument.

Published shortly after Schrag’s monograph, Laura Stark’s *Behind Closed Doors* is an ethnographic study (again from the US) based on observations of IRB meetings and interviews with IRB members, with some elements of a historical exploration. The historical account focuses on the origins of the method for making decisions about research ethics: group review by experts required to reach a consensus. After over 60 years of using this approach, it may appear to be the most reliable and overall ‘best’ way of making research ethics decisions. Yet group reviews are also a consequence of a particular historical configuration: IRBs are “direct descendants of the Clinical Research Committee that met inside NIH’s Clinical Center starting in the 1950s” [*op. cit*., p. 157]. Stark also argues that rather than having group review imposed on researchers by outsiders (such as bioethicists or activists, as the story often goes), it was primarily researchers and NIH leaders who developed, justified and expanded this mechanism “as a technique for promoting research and preventing lawsuits” [*op.cit.*, p. 8].

Core findings from Stark’s ethnographic work concern the IRBs’ methods for making decisions. For example, board members appeared to be reading “researchers’ application documents like tea leaves for signs of good character” (p. 6). Most notably, the researcher’s apparent attention to detail was translated into judgments of his or her professional competence and trustworthiness (pp. 17–18). Board members used “warrants” to justify their recommendations and although some pertained to matters of fact and professional expertise, a surprising number came from their private lives. The people who featured there came to serve “as stand-ins for research participants”, thus reinforcing the biases of IRB membership (p. 14). IRBs typically invoked local precedents (p. 47 onwards) in their decision-making processes as opposed to abstract ethical rules. This, Stark argued, ensured efficiency and internal consistency of decisions over time, but was also the source of the much-lamented variability of decisions across IRBs. (Of course, we need to acknowledge the critical stance of “Behind Closed Doors”, meaning that it draws more attention to unacknowledged and problematic aspects of the system as opposed to its strengths and good processes.)

The empirical and critical explorations of research approvals in published papers have a rather limited overlap with the work of Scrhag and Stark. No relevant literature review seems to have been conducted, whether systematic or traditional. We offer a preliminary summary of key themes and approaches, with a focus on the last 20 years.

“Horror stories” appear to be one of two main ‘genres’ in the literature, with the term first used by Bradford Gray in 1978 of the “sad tales told by scholars in their letters to the National Commission and in their testimony at the IRB hearings of 1977” (p. 161) [3]. Mary Dixon-Woods et al. call them “wounding encounters” [5]. More neutrally, these are case studies of challenging ethics review processes, due to time delays and inconsistencies of decisions across committees; [6–11] bureaucracy, form filling, paperwork and over-concern with minor language and grammar issues; [12–14] lack of appropriate expertise, which may lead to inappropriate rejections, criticisms and recommendations; [15] blocking of important research [16] or compromising its rigour, e.g. through negative effects on recruitment [17]. Mounting costs are also a source of frustration. Most strikingly, Kano et al. estimate personnel costs of obtaining the 84 approvals for their multi-site project at US$121,344 [18] (note, however, that the system for multisite projects in the US has now changed).

Some of those publications are strongly emotionally charged. Mentions of “horror stories” and “wounding encounters” are also typical elements in polemical texts of scathing criticism and radical dismissal of the value of the existing systems of ethics review, especially if applied outside of biomedicine, such as to social sciences research (e.g. see Dingwall and to some extent Derbyshire [19, 20]). Many publications describing significant challenges, however, aim to remain objective and constructive, giving specific recommendations for improvements rather than questioning the overall value of the system.

There are also studies which, while arising from a background awareness of the challenges around ethics approvals, go straight into exploring and evaluating models for optimising the work involved. Recent research of this type concerns, for instance, the introduction of an ethics officer, [21] the use of structured tools to evaluate the work of RECs/IRBs, whether through self-assessment or researcher assessment, [22, 23] models for simplifying standard operating procedures, [24] and approaches built around specific conditions and enabling better separation between the scientific and ethical appraisal within the ethics review [25].

More broadly, audits and evaluations of RECs/IRBs, typically survey-based, appear to be the other leading genre in the literature. This may be a historical path dependence dating back to 1975, when the National Commission for the Protection of Human Subjects of Biomedical and Behavioral Research (the commission which created most of the foundations for regulating human subjects research) commissioned the original survey of researchers and IRB members (see p. 63 onwards in Schrag [3]). In the last decade, topics include the overall availability and profile (composition, arrangements for their work, etc.) of IRBs and RECs, primarily in areas where ethical regulation of research is new; [26–32] comparisons of committee members’ and researchers’ views on ethical issues in complex areas; [33–35] practices, policies and requirements of IRBs/RECs; [36–38] committee members’ knowledge of and attitudes to ethically and legally complex topics [39, 40] and researchers’ perceptions of the review process, outcomes and impact [2, 41, 42].

The literature described above makes little explicit use of theory. In contrast, there is an identifiable niche of work on ethics approvals drawing on theory from the social sciences [5, 9, 43–45]. It problematises the foundations of the new regimes of control and warns against their dangers, particularly in the context of the social sciences. Narrower topics are also explored. For instance, Dixon-Woods et al. look into the social functions of REC letters, including ‘stabilising “what is ethical” in a world where ethical matters are in fact indeterminate’; providing institutional displays of “care, thoroughness and thoughtfulness”; and structuring the relationship between the REC and applicants, reinforcing a ritualised supplicant-authority relationship [5]. Or, against expectations that REC decision-making is yet another impersonal process of a modern bureaucracy, Hedgecoe argues that local knowledge, trust and “facework” (roughly, the direct face-to-face interactions between applicants and committee members which serve to build trust) are surprisingly important for the decision-making processes of local RECs [44].

Some recent trends in the literature can also be identified. There is a fast growing global dimension to it (see, for instance, [46–49]; about a fifth of the 100 most recent publications retrieved by “ethics research committees” as a major heading in PubMed, Jan 2018, were from developing world countries or countries with smaller research communities). There is also attention to ethics approvals challenges and solutions for newer or newly popular study types, such as participatory research or co-design studies, [50–53] biospecimens and biobanking studies, [54–56] digital health research [57–59] and research in disaster and developing world contexts [60–65].

Finally, there are red threads of issues which do not become the focus of texts but are picked and interweaved in much of the critical literature. It often reminds us that ‘there is no single “right” answer to (most) ethical issues’ (p. 795); [5] that what constitutes ethics, ethical practice and ethical decisions is contested, contextual, theory-dependent, subjective, personal, relational, evolving … (e.g. in 2, 9, 43). In contrast, what filters down to ethics review forms and processes is often an “impoverished ethics” (Carl Schneider quoted in Schrag [3], p.3). The shift of power from scholars to administrators and the rise of a bureaucratic and self-serving “machine” are lamented. Appeals are made to trust researchers and their professionalism more and enable them to take more responsibility for the ethical decisions they make. Attention is repeatedly drawn to serious flaws in or lack of relevant definitions, classifications and boundaries, which have profound impact on what is being regulated and resulting, for instance, in journalists conducting (and being praised for) investigations which social scientists would never be permitted to do or the state performing observations that are far more secretive and sinister that any researcher would ever consider engaging in.

Against this backdrop, the empirical aspect of our paper is a case study of ethics and governance approvals, with a focus on quantifying aspects of the process, one of which does not seem to have been quantified before (the number of “approvers” involved). Although some of the findings are striking, we did not have the experience of a “horror story” or “wounding encounter”. We did *not* start an audit because we were frustrated. Our study arose out of a table we kept in the project Master File to avoid excessive printing and to justify not printing (communication concerning project approvals must be printed and stored in a physical Master File). If it were not for this table, we would not have paid any further attention to the approvals process once we had gone through it.

This is one of the important points we want to make: much of the burden of ethics and governance approvals and the complexity of the system remain too vague or subliminal to be perceived clearly, like the underwater part of an iceberg. We fail to change course in steering ahead, as we can hardly discern what lies beneath, and we end up damaging the integrity of our research.

Inevitably, by virtue of the position we are taking, as researchers who have found that, largely unknowingly, we have been part of a rather inefficient process, our presentation of issues may feel or be one-sided. We acknowledge that there are many more sides to the story that need to be heard before we arrive at a balanced view of the advantages and disadvantages of current systems. This is one of them.

## Main text

### The substantive project (study being approved)

The substantive project whose ethics and governance approvals we are discussing was a relatively small mixed methods study, “Prepared to Share?” [1] on patient data sharing in complex conditions, advanced and progressive disease and/or at the end of life, conducted by one Primary Investigator (PI, SB) and one Research Associate (RA, MP). It was an evaluation of the development, implementation and outcomes of a local Electronic Palliative Care Coordination System (EPaCCS) nested in broader research on attitudes to patient data sharing. As the EPaCCS service development project was underway while the study was being carried out, the study was contributing to the EPaCCS project, but the two remained largely independent. Primary data collection was carried out between 2013 and 2015 in Cambridgeshire & Peterborough Clinical Commissioning Group, England’s second largest CCG. It included:

1. *qualitative interviews:* 40 with 44 primary participants – project developers; General Practitioners (GPs) in their role as data sharing initiators; data sharing recipients including palliative care specialists, out-of-hours, hospital and 111 staff (111 is a non-emergency medical helpline in England, Scotland and parts of Wales); patient and carers; key informants from the local area; and a practice administrator. (There is a discrepancy between number of interviews and participants as some patient and carer interviews involved more than one person and the two project leads were interviewed twice; paper in preparation);
  No question touching on end of life care was included in the interview schedules for patient and current carers. The focus was fully on data sharing, although end of life issues were discussed when raised by interviewees.
2. *questionnaire* concerning GPs’ and Practice Managers’ (PMs) familiarity, engagement with and perceptions of patient data sharing (405 responses, 64% response rate); [66]
3. *ethnographic observations*, mostly from the process of developing the data sharing system, but also from use of the data sharing tools in healthcare settings;
4. *document analysis,* using reports, meeting minutes and email communication of the clinical service development project.
  Three papers have been published from the study, [66–68] with more in preparation.
  While we could have attempted to represent the study as an evaluation (an exempt category), as its primary aim was to evaluate a service, we decided against this course of action. There were vulnerable individuals in our intended sample, regardless of how a study is classified relative to a debatable and often arbitrary distinction. We wanted to benefit from expert feedback in a full review (as opposed to a proportionate one) and also ensure that the team was fully protected, should untoward events occur. The benefit of saving time was also uncertain. Exempt studies need a form of approval, be it minimal, as it is advisable to have them confirmed exempt through a formal letter (which may be required by journals before considering publication). In addition, not going through an ethics approval would have still meant going through all other governance approvals, which are often facilitated and/or expedited if preceded by an ethics approval.

### The project team

As the relative ease or difficulty of going through ethics and governance approvals is affected by the team members’ research experience, we are adding a brief section to that effect. SB is an experienced, internationally recognised palliative and end of life care researcher, university lecturer and GP, with all his senior level career in the locality of the study whose approvals we discuss. MP is a late-early to mid-career researcher, with some of her education and research experience strongly ethics-focused (an MA in Philosophy and Ethics of Mental Health, for which she even wrote coursework on the value of research ethics committees, and several years of experience on a research programme in “values-based practice” as complementary to evidence-based practice).

### The system used for study approval and its relationship to HRA approval

Study documentation was submitted through the Integrated Research Application System (IRAS), the UK’s single electronic system for applying for permissions and approvals for health and social care research. It was through the Coordinated System for gaining NHS Permission (CSP), a simplified system available primarily to studies on the National Institute of Health Research (NIHR) Portfolio, phased out in March 2016. It is important to note that the processes we used have now been modified. Below we present the key claims concerning the changes made. We give the new system the benefit of the doubt but maintain that it will not resolve many of the issues we encountered and, under individual findings, explain why. We also excluded our data on time delays, as much as they were a substantial concern (including 7 months for National Institute of Health Research Portfolio application and 7 weeks for minor ethics amendments). Dealing with delays was one of the strongest motivators for introducing the new system and a parameter showing improvements as a result of it.

The new Health Research Authority (HRA) approval process for England was rolled out on 11th May 2015. HRA Approval is represented as a single “radically simplified” approval system for all studies involving the NHS [69, 70]. At the heart of the reform has been closer integration of the systems for REC review and NHS R&D permissions. This would remove the need for repeated R&D permissions, as granted by each individual NHS organisation involved in a study. The changes are designed to reduce system complexity, timelines and costs; eliminate duplication; make the UK a more attractive place for health research and free up of capacity for research delivery and implementation. [70] In June 2016, HRA announced a backlog of 3000 amendments [71] – pending approvals of changes to existing projects. Bedding-in difficulties can be expected with any significant reform and at least the above seem to have been resolved. A performance report from September 2017 [72] showed that HRA Approval timelines (after final REC favourable opinion) reduced significantly between November 2016 to March 2017, after which they levelled out. The report also stated, however, that only 14% of the current HRA caseload were studies actively processed by the HRA team. The remaining 86% were awaiting other approvals or a response from the applicant [*op. cit.*]. There is something amiss in a system which controls the outcome of less than a seventh of its cases.

### The approvals audit

This audit-type study arose out of a summary table of approvals communications maintained for the Master File of “Prepared to Share?”. Our first recorded communication about ethics and governance approvals is from February 2013. The last document was issued in February 2016. (Chronologically, some of the approvals were granted at a time when HRA Approval was already functional. However, the new system was phased in gradually and our study was fully approved under the old system.) During the most intense period of approvals we had no intention to formalise the emerging evidence, i.e. bias attributable to intentional observations of system inefficiencies is unlikely. Findings are based on records kept by the RA (MP) only.

#### Eighty-nine individuals (a conservative estimate) have been involved in the study approvals

The study team have communicated with 81 named individuals for research ethics and governance approval purposes through email, telephone or in person, with 8 unnamed individuals mentioned in communications as having provided further advice. These numbers leave out seven categories of individuals and contributions of which we are aware, primarily because communication has been too indirect (such as with staff who processed criminal records checks) or not strictly required (such as with gatekeepers who do not have the authority to grant or withhold permissions formally but could facilitate or block the study progress in other ways). There are also individuals involved in the permissions process who remain invisible to researchers. (In some cases, different individuals performed the same role, if working as part of a team or if post-holders changed. However, the same individual could also perform several roles.)

Thirty-five out of the 44 participants interviewed were health professionals, decision makers and project developers and 9 were patients or family members. This corresponds to roughly two approvers per participant, with all the necessary caveats added about the meaningfulness of such statistics. In one hospital, 10 individuals and 101 exchanges outside the supposedly “one-stop” IRAS platform were needed to interview 4 consultants, a group whose decision-making capacity is hardly a matter of debate. Table 1 provides further details of the distribution of individuals across organisations.

**Table 1:** Individuals and exchanges involved in the ethics and R&D approvals of the substantive study

Setting/ Type of approval	Number of named individuals from organisation	Number of recorded exchanges outside IRAS	Number of pages of text generated from exchanges	Number of interviewees
Local R&D approval and study assurances, in conjunction with Clinical Research Network (CRN). Primary outcomes - decisions on CRN support and approval of access to GP practices	15	110	63.5	9 patients and carers 14 GP practice staff
NHS Research Ethics Committee (REC)	19	45	9.5	9 patients and carers
Sponsor (University)	17	67	31.5	NA
Research passport	No separate organisation	37^a^	14.5^a^	NA
Organisation 1	10	101	30.5	4 consultants
Organisation 2	4	78	23.5	3 nurses
Organisation 3, process not completed^b^	2	12	5	–
Organisation 4, process not completed^b^	6	27	9.5	–
Organisation 5, process not completed^b^	2	14	5.5	–
Other (e.g. funder, original employer)	6	included above	included above	NA
TOTAL	81	491	193	30^c^

^a^ As the study was associated with a service development project which was not taken up as expected by Organisations 3, 4 and 5, we decided against conducting interviews with their members of staff. The complexity of R&D approvals was a contributing factor

^b^ Some of the communication was conducted from a now closed institutional email account. We had recorded the contents of exchanges, but had not saved the emails. As a result, these particular figures are an underestimate

^c^ Patients and carers are included in two boxes, as they were a core concern for two approvals. The remaining 14 interviewees (above the total of 30 in the table) were not covered by specific rules

Reducing the involvement of individual NHS sites would still leave 59 individuals granting formal approvals. Many of those staff whose signatures will no longer be required will nonetheless be enabling a study’s conduct at a site – a lesser gatekeeping hurdle to overcome, but still an important one.

#### Four hundred and ninety-one exchanges with those individuals were recorded outside the Integrated Research Application System (IRAS)

We have recorded 491 exchanges with these 89 individuals, generating 193 pages of email text, *excluding* attachments. These attachments, whose total volume we have not quantified, could contain key information (e.g. amendments requested) or be voluminous (e.g. one of the first emails we were sent by the local R&D team had 76 pages of attachments; a core set of study documentation which we have been sending out numbered 78 pages). In theory, the Integrated Research Application System should act as the platform where all information necessary for research approvals is requested and provided. In practice, information not requested in IRAS forms becomes relevant, especially with non-standard studies; documents are uploaded on IRAS but are not accessible to certain groups; or documents are managed as hard copies and only sent to the study team who then need to pass them on (REC favourable opinion letters were a case in point).

While human inefficiencies on both sides will have had some impact (however we may want to minimise them through self-serving biases), it will be problematic to explain the full volume of correspondence external to the IRAS system through the incompetence of its users, whether our or of our colleagues managing the approvals. We recognise that IRAS is a living platform undergoing continuous improvement, but its current version is still largely the same as the one we used. There is more that an integrated research application system should enable to deserve its name.

#### The 31-page IRAS application and key study documentation appeared to have been read in detail at least 13 times for one-and-a-half pages of mostly technical suggestions

The 31-page IRAS application and accompanying documentation have been read in detail at least 13 times – a conservative estimate based on instances when informed questions were asked or comments provided (6 REC, 4 local R&D, 2 Sponsor, 1 specific NHS site). We cannot estimate the number of people who have read specific sections from the study documents (a typical set consisted of 78 pages and a minimal set of 23 pages). The total written concerns raised by these 13 people amounted to just one-and-a-half pages of text and addressed almost exclusively minor omissions or clarifications of research procedures. We introduced more changes to our study documentation as a result of consulting the study Lay User Group than as a result of the ethics and R&D review processes.

#### The most significant ethical issues we experienced arose outside of the NHS ethics committee mandate

The most significant ethical issues we experienced in conducting the research related to interviews with members of the service development team working on the data sharing system evaluated as part of the study. The interviews revealed unexpected sources of dissatisfaction and internal conflict. As both of us are also members of the service development team, devising informative yet sensitive ways of feeding back findings was challenging. There was information we agreed to withhold at this stage and re-consider for publication in the future, as it was deemed potentially damaging to team relationships.

It is hard to tell whether the risks of stirring intragroup conflict could have been picked more efficiently if research on staff were formally reviewed as part of the NHS ethics review process. Hindsight will often mislead us in answering such questions. No matter how thorough an ethics review, it will never be able to cover the whole complexity, risks and eventualities arising in the real world. But it is worth noting that NHS ethics review does not formally consider research on healthcare professionals and non-NHS staff, even though we were required to submit information on this aspect of the study for ethics approval, by virtue of it being part of the broader project.

#### The study was often unclassifiable under existing frameworks, leading to lengthier debates and timeframes

There were four types of classification difficulties associated with this study which generated protracted and recurrent discussions and delays: 1) the study had both research and evaluation aspects; 2) it involved both patients and health professionals; 3) it combined traditional research and service development elements and was set both within a University and a healthcare setting; and 4) it was funded through an open competition in a geographically confined area, i.e. an open competition within a closed competition. The decision pathways and outcomes for the “pure cases”, here combined in a single study, clashed. For instance, while ‘patients’ and ‘research’ (unless on staff) necessitate NHS REC approval, ‘evaluation’ and ‘staff’ are exemption criteria. Or, since the grant, being primarily for service development, was held by an NHS-related organisation rather than the University as a research grant, sponsorship responsibilities could not be attributed unequivocally. Finally, the research project was funded in two open competitions within narrow geographical and organisational boundaries. This led to protracted discussions concerning financial and infrastructure support from the Clinical Research Network, only available to studies which have undergone high quality peer review and been funded through an open competition.

### Implications of the findings

Our findings confirm both empirical findings from previous research on ethics and governance approvals and impressions which are shared informally amongst many researchers working in human subjects research. These concern system complexity, duplication, excessive paperwork, attention to trivial detail in applications while missing core ethical issues, and classification difficulties with significant consequences for the approvals process and outcomes.

We also suggest two new points. The first is that the size of the approvals machine and the nature of the work of its individual parts are largely unknown even to people who are working in it, including those working to reform it. Colleagues and reviewers from within the system have been struck by the figures (as we were) and even doubtful of our data. This is largely because the ethics and governance approvals system is a single whole only by virtue of its outcomes – permissions for a study to proceed. Other than that, it is at best a collage, at worst a ‘bag’ of elements belonging to many different organisations where only some are well connected to others.

The second point also concerns issues around visibility and knowability. While many researchers may be experiencing ethics and governance approvals processes as burdensome and time- and resource-consuming, much of that burden and investment remains under the radar. As we were moving through the hoops of the approvals process, we felt we knew how to navigate it, believed we were managing it well, and found the staff and signatories on the other side unfailingly helpful, even if not always efficient. Our frustrations were only occasional and fleeting. We were completely unprepared for some of the figures generated by the case study. The scale of the work involved is largely invisible when done piecemeal. It is worth re-emphasising that the work described here excludes the core task of obtaining ethics and R&D approvals – completing the paperwork (such as the IRAS form) and preparing the accompanying materials (such as information sheets). Also as mentioned, this case study was based on records held by the RA only, document attachments were not counted in, and estimates as a whole were conservative. The impact on the cost of studies and loss of opportunity to focus on the substantive research is enormous, even when considering an incomplete picture.

### Steps towards a truly “radically simplified” system

Even though HRA Approvals seems to be achieving some improvements, we argue that more needs to change before researchers in England experience the system as “radically simplified”. We propose six priority steps for enabling such change. We believe that these steps are likely to be generalisable to other contexts, as many of the challenges they aim to address are shared, as the literature review has demonstrated. In addition, our proposals are predicated on flexible and dispersed working processes, which are becoming increasingly natural with the growing sophistication of information technologies.

#### Support the development of a broad range of customised research ethics and governance templates rather than rely on generic, predominantly clinical trials orientated, templates

Templates for soliciting ethically relevant information for health research, at least in England, are typically modelled on templates for clinical trials for investigational medicinal products (CTIMPs). While screening questions from other ethically charged areas are also asked, for instance concerning research on vulnerable groups (e.g. children, prisoners, individuals lacking mental capacity), utilising human tissue, involving radiation, etc., these questions are limited. Any health and medical research involves its particular ethical issues. In our case, issues around palliative care research, evaluations of active projects and data sharing received limited or no attention, not least because the proformas through which the information was collected were generic. Ethical and regulatory challenges will be far more effectively captured if templates are customised to different study types, through endeavours informed by domain experts, bioethicist and patients.

There are examples of successful introduction of subject-specific templates for IRBs and their positive impact [73]. There are also settings where health research informed by the behavioural and social sciences and the humanities is reviewed by committees within the respective academic departments, where forms are likely to be more appropriate. Needs for improving template relevance are not universal, but sorely needed in some contexts. Inevitably, such work will create its own challenges, most notably template proliferation and version control. But unless we become more accommodating of the huge variety of studies being conducted, we will continue to stretch and shrink studies to the Procrustean bed of CTIMPs.

#### Improve study classification, particularly in view of the growing number of innovative and mixed study types

The liminal nature of our study on at least four criteria (e.g. research – evaluation, study of patients – study of health professionals, etc.) was a cause of uncertainty and delays in decisions. To have a system that is equitable and efficient across study types, we need new and expanded decision pathways which can accommodate the growing number of non-typical and multi-method studies.

Possibilities for omissions of ethical and governance challenges based on typological distinctions should also be reduced. Currently, service evaluations and NHS employee research are considered to pose low ethical risks. This is an oversimplification. Most likely, there are no essential typological differences between research and evaluation studies in the ethical challenges they pose. There are simply lower litigation risks associated with studies classified as evaluations. Apart from missing relevant ethical considerations, the current research-evaluation distinction is a source of unequal treatment of projects (i.e. when two very similar projects go down different routes, one of evaluation, the other of research) and is open to manipulation. Even the most responsible amongst researchers are tempted to consider if we can “pass” a study as an evaluation to avoid the complex NHS approvals process.

#### Link with associated processes for assessment, feedback, monitoring and reporting

The ethics and R&D approvals of a study are only one of the spaces where the methods, design, supporting materials, staff competence, the adequacy of funding and other features of a study are reviewed. Funders, the scientific reviewers whose feedback they solicit, patient and public involvement (PPI) groups and research support services often review significantly overlapping, even the same, documentation. On the monitoring and reporting side, funders, ethics and R&D officers, university administration, impact and pubic engagement officers, PPI groups also have overlapping needs and requirements. While the availability of alternatives can ensure the robustness of the system, creating opportunities to rectify omissions not identified previously, it also creates wasteful redundancy and duplication of effort. Better alignment of the work of these different organisations and networks will lighten up the overall “meta-research burden”, i.e. all those tasks associated with the research support infrastructure as opposed to the substantive scholarly work.

#### Invest in a new generation IT system

If HRA Approval, or any ethics and governance approval system, strives to be radically more efficient, work on developing the underpinning IT system should be prioritised, with an ambitious vision. Until all study documents are located in one place and easily searchable, voluminous email exchanges outside of the system will continue. Easier access to full study information will enable de-localising and de-synchronising aspects of the approvals process that need not be geographically and temporally fixed. For instance, comments on the ethical aspects of study protocols or the quality of patient information materials may be more efficiently provided by appropriately qualified but geographically dispersed individuals rather than a local REC committee. Broader lay representation will also be facilitated in this way. Apart from improving efficiently, such broadening of participation could also enhance the quality of ethical debate.

Furthermore, rather than each PI sending to non-university bodies credentials which are identical for every study within a university (such as insurance documents, which also periodically expire and are renewed, resulting in further communication), a university can have “an institutional legal, ethical and governance profile for research” and access to all of its studies on a shared system for approvals. Similarly, rather than repeatedly attaching the same CVs and Good Clinical Practice certificates, PIs and RAs can have personal “ethical and governance awareness profiles”, also linking to established platforms such as ORCID, ResearchGate, LinkedIn or Convey (a platform for conflict of interests disclosure [74, 75]). Finally, the non-proprietary, non-sensitive information concerning studies and their ethical credentials can be made available to the public through the same system using different (or similar) viewing rights. Study participants in particular should have easy access to such documentation and opportunities to report on the ethical conduct and adverse effects of being in a study.

#### Enhance system capacity through increasing online participation and training

One of the reasons for the slow ethics and R&D approvals process is that it is highly localised. In the case of NHS ethics reviews, this is still focused around an ethics committee that meets physically to discuss studies and thus has a limited membership, often worryingly professionalised, and a high workload. If we broaden the number of potential participants in the review processes through a sophisticated IT system and accredited training, we can dramatically increase the capacity of the system. Some possible directions for recruitment efforts include medical charities and patient groups, whose involvement in research has been growing steadily in recent years, postgraduate students in ethical disciplines, and, perhaps most importantly, past research participants. Involving the latter, however, requires us to step up efforts to keep participants informed of the outcomes of studies, recognise their contribution, and nurture their willingness to support research in a variety of ways.

Perhaps, in the not too distant future, existing Research Ethics Committees will turn into local training and advice centres, while much larger ethics review teams do most of the work online.

#### Encourage researchers to quantify the approvals process they are going through

Finally, we hope that more colleagues will be willing to undertake work similar to the one presented here and that more structural support for bigger and more rigorous studies, as opposed to single case studies, can be provided by funding bodies. If researchers can offer evidence concerning ethics and governance approval processes, rather than primarily express frustration amongst ourselves, we will have much stronger arguments to insist on radical improvements and better ideas to enable them.

## Conclusions

The broad variety of stakeholders who experience the impact of burdensome research ethics and governance approvals processes – researchers, R&D staff, health professionals involved in research, research participants, funders, administrators, etc. – have a responsibility to facilitate and demand substantial improvements of the system. Unless we contribute to such change, we will continue to uphold principles of research ethics in a way which is deeply unethical, by virtue of its wastefulness on the one hand and impoverished ethics on the other.

## Abbreviations

HRA: Health Research Authority
IRAS: Integrated Research Application System
NHS: National Health Service
NIH: National Institutes of Health
R&D: Research and development
REC: Research Ethics Committee
